# A case report of *PGAP2*-related hyperphosphatasia with impaired intellectual development syndrome in a Chinese family and literature review

**DOI:** 10.3389/fped.2024.1419976

**Published:** 2024-12-02

**Authors:** Yijun Pan, Bin Ren, Lijuan Chen, Qiang Li

**Affiliations:** ^1^ Department of Pediatric Neurology, Guiyang Maternal and Child Health Care Hospital, Guiyang, China; ^2^ Department of Genetic Counseling, Shanghai Nyuen Biotechnology Co., Ltd., Shanghai, China

**Keywords:** PGAP2 variants, hyperphosphatasia with impaired intellectual development syndrome, epileptic spasms, facial malformation, ACTH treatment, pyridoxine

## Abstract

Recently, mutations have been identified in six genes (*PIGA*, *PIGY*, *PIGO*, *PGAP2*, *PIGW* and *PGAP3*) encoding proteins in the Glycosyl phosphatidylinositol(GPI)-anchor-synthesis pathway in individuals with hyperphosphatasia with impaired intellectual development syndrome(HPMRS). Reports involving the rare pathogenic gene, post-GPI attachment to proteins 2 (*PGAP2*) are quite limited. In this study, we reported two patients with *PGAP2* variants related neurodevelopmental disorders from Asian population. The proband, onset of epileptic spasms at 5 months, concurrently with global developmental dalay, facial malformation and elevated alkaline phosphatase. His younger sister, onset of epileptic spasms at 2 months, having similar clinical features as the proband. Their phenotypes are consistent with *PGAP2* related diseases. The two missense variants [c.686C>T (p.Ala229Val) and c.677C>T (p.Thr226Ile)] in *PGAP2* gene found in this family were segregation with the disease, while c.677C>T (p.Thr226Ile) was a novel variant. All the two patients showed a positive response to ACTH treatment and high-dose pyridoxine. In summary, this study contributes to expanding the pathogenic variant spectrum of *PGAP2* related HPMRS, and provides new insights into the treatment.

## Introduction

Glycosylphosphatidylinositol (GPI) anchoring is a post-translational modification that attaches proteins to the plasma membrane, playing a role in protein sorting and transport. GPI anchoring proteins are highly conserved across eukaryotes, with mammals possessing over 150 GPI-anchored proteins (GPI-APs). Including receptors, adhesion molecules and enzymes (1). Defects in six genes involved in the GPI-anchor synthesis pathway-*PIGV* (MIM:610274), *PIGO* (MIM:614730), *PIGY*(MIM:610662), *PIGW*(MIM:610275), *PGAP3*(MIM:611801) and *PGAP2* (MIM:615187) -are associated with hyperphosphatase with impaired intellectual development syndrome [HPMRS (MIM 239300)](also known as Mabry syndrome) (1). HPMRS is a rare autosomal recessive genetic disorder, affecting both males and females, characterized by intellectual disability, hypotonia with poor motor development, speech disorder, and elevated serum alkaline phosphatas, reflecting a broad phenotypic spectrum of disorders that include ID and seizures (2–5).

Recent studies have identified that mutations in *PGAP2* are linked to HPMRS-3, which displays phenotypic overlap with the broader HPMRS category. HPMRS-3 is inherited in an autosomal recessive manner, with both homozygous and compound heterozygous variants reported, often in the children of consanguineous parents (2, 6–9). *PGAP2* encodes a Golgi/ER-resident membrane protein involved in fatty acid remodeling of GPI-APs in the Golgi, where it involved in reacylation with stearic acid, a saturated fatty acid (10, 11). Fatty acid remodeling is essential for ensuring proper association of GPI-APs with specific membrane domains known as lipid rafts or lipid microdomains (11, 12). Function study have indicated that *PGAP2* deficiency caused transport to the cell surface of lysoform GPI-APs that were easily cleavage by phospholipase D (2, 10, 13). However, reports on the *PGAP2* gene remain limited, with only 16 pathogenic variants in 25 patients documented (2, 6, 8, 9, 13–15).

In this study, we report a Chinese family with two children diagnosed with recessive HPRMS-3 through genetic testing. Both siblings are compound heterozgous for two *PGAP2* missense variants (c.686C>T (p.Ala229Val) and c.677C>T (p.Thr226Ile). To our knowledge, this is the first report about *PGAP2*-related neurodevelopmental disorders in East Asian population. The study was accompanied by a comprehensive literature review about the genotypes, phenotypes of the condition. Given the limited number of reported cases to date, an expanded clinical delineation of HPMRS-3 and insights for targeted therapeutic interventions would be greatly enhanced by the identification and longitudinal assessment of additional cases.

## Materials and methods

### Subjects

A 4 year and 5 month old boy (proband, II-1) and his younger sister (II-2) were admitted at Guiyang Maternal And Child Health Care Hospital. Tiro whole exome sequencing (trio-WES) was performed on the proband and his parents. The younger sister was also enrolled in this investigation to elucidate the genetic inheritance pattern. This study was approved by the Medical Ethics Committee of the Guiyang Maternal and Child Health Care Hospital and the written informed consent from genealogy parents was obtained.

### Whole exome sequencing and data analysis

The clinical whole exome sequencing (WES) of the proband and their parents was performed to determine their genetic etiology. Genomic DNA was extracted from whole blood samples for library preparation using Hieff NGS OnePot Pro DNA Library Prep Kit for ILLUMINA (Yeasen, China). The xGen Exome Research Panel probes (IDT, USA) were utilized to capture the exon region following the manufacturer's recommendations. The raw data was sequenced on NovaSeq6000 platform (Illumina). Reads were then aligned to the hg19 human reference genome (GRCh37) using BWA MEM (v0.7.17). Subsequently, PCR and optical duplicate marking were performed using Genome Analysis Toolkit GATK(v4.1.4.0) and local realignment around indels and base quality score recalibration were carried out by GATK (v3.8.1). Finally, the variants were identified using GATK HaplotypeCaller 1 (v3.8.1). Variants were annotated by Annovar and the pathogenicity of candidate variants wase valuated according to American College of Medical Genetics and Genomics(ACMG) guidelines (16, 17).

### Sanger sequencing

Sanger sequencing was performed on the two siblings and their parents. The primers design was accomplished using primer3 and synthesized by Beijing Tsingke Biotech Co., Ltd. (Beijing, China). The forward and reverse primer sequences were as follows: Forward 1: 5'-AAGCTGCAGAGTGATCAGACAG-3'; Reverse 1: 5'-GAAGGCCAGAATGG TATCGTGT-3'; Forward 2: 5'-ATCCACAGGCGGTCAT GAGT-3'; Reverse 2: 5'-AGCCTTGGCCTACACCCTTC-3'. Polymerase chain reaction(PCR) was performed using a ABI2720 (Applied Biosystems). The amplified products were sequenced using ABI3730 (Applied Biosystems).

## Results

### Clinical overview of the pedigree

**Figure 1:**
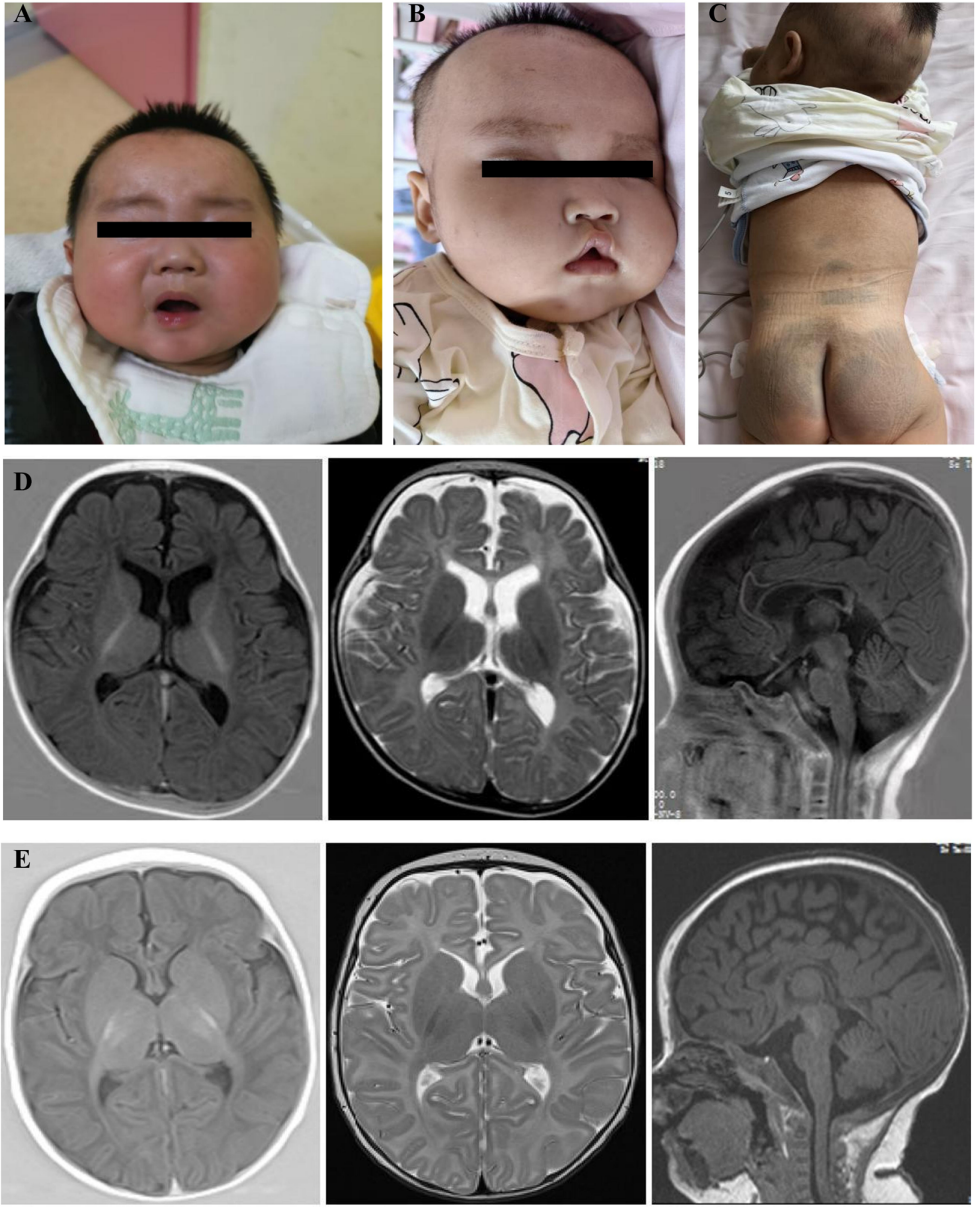
Facial features and brain MRI results of the proband and his younger sister. **(A)** Facial features of the proband at 19 months of age: thick eyebrows, hypertelorism in both eyes, and collapsed nasal bridge. **(B)** Facial features of the proband's younger sister at 2 months of age: thick eyebrows, collapsed nasal bridge, tented upper lip, and downward-facing corners of the mouth; **(C)** Large Mongolian patches on the dorsum and rump of the proband's younger sister **(D)** Proband, 5-month-old cranial MRI: thin corpus callosum, slightly enlarged lateral ventricles, mildly widened extracerebral spaces in bilateral fronto-temporo-parietal lobes, transverse axial T1W, T2W, and sagittal T1W. **(E)**Proband's younger sister, 2-month-old cranial MRI: hypoplastic corpus callosum, transverse axial T1W, T2W, and sagittal T1W.

**Figure 2:**
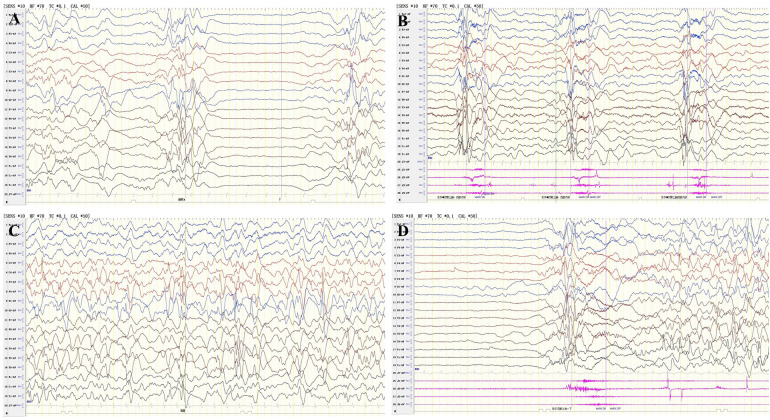
VEEG results of the two patients. VEEG of 5 months of age of the proband, **(A)** Interictal period: hypsarrhythmia with burst suppression; **(B)** Ictal period:clusters of epileptic spasms. VEEG of 4 months of age of the proband's younger sister, **(C)** Interictal period: hypsarrhythmia; **(D)** Ictal period: epileptic spasms.

The proband, a male infant, presented with clusters of epileptic spasms at the age of 5 months. The patient had no significant medical or perinatal history and exhibited global developmental delay. Family history: The younger sister displayed similar manifestations. Physical examination revealed thick eyebrows, hypertelorism in both eyes, collapsed nasal bridge (Figure 1A), and hypotonia in the limbs. The results of routine blood tests, liver and kidney function tests, electrolyte levels, blood amino acids, acylcarnitine levels, and urine organic acid analysis were within normal limits. However, there was an elevation in serum alkaline phosphatase (ALP) levels to 789 U/L. Brain MRI revealed a thin corpus callosum, slightly enlarged lateral ventricles, and mildly widened extracerebral spaces in bilateral fronto-temporo-parietal lobes (Figure 1D). VEEG monitoring showed bilateral posterior head dominant hypsarrhythmia with burst suppression, bilateral posterior head dominant fast wave rhythmic discharge during sleep episodes, as well as clusters of spasms (Figures 2A,B). The administration of ACTH significantly reduced seizures; however, there was a recurrence of epileptic spasms and the emergence of atypical absence seizures. Despite the ineffectiveness of various antiseizure medications (ASMs), the gradual addition of high-dose pyridoxine provided relief from seizures. At 2 years and 10 months old, he exhibited impaired independent sitting, active grasping, and speech abilities. Subsequent repeated examinations revealed a significant increase in his ALP levels (789–3380 U/L).

The proband's younger sister, a female infant, presented with clusters of epileptic spasms at the age of two months. Physical examination revealed thick eyebrows, collapsed nasal bridge, tent-shaped upper lip, large patches of Mongolian spots on the back, buttocks and trunk (Figures 1B,C), and low muscle tone in the limbs. There was no significant history during perinatal period. Global developmental delay was observed. Family history showed similar manifestations in the elder brother. Blood routine tests, liver and kidney function tests, electrolyte levels, blood amino acids and acylcarnitines as well as urinary organic acid levels were normal; ALP level was 619 U/L. Brain MRI revealed hypoplastic corpus callosum (Figure 1E). VEEG monitoring indicated hypsarrhythmia and spasms (Figures 2C,D). ACTH treatment significantly reduced epileptic spasms but seizures recurred until high-dose pyridoxine was added to alleviate symptoms gradually over time. The patient is currently five months old with stable head control but poor sound/object tracking ability; he is unable to turn over or grasp objects actively.

The two children have no other siblings. No other family history of neurological disorders, behavioral issues, or seizures was reported in this family. Both parents were healthy and nonconsanguineous.

### Genetic analysis and literature review

**Figure 3:**
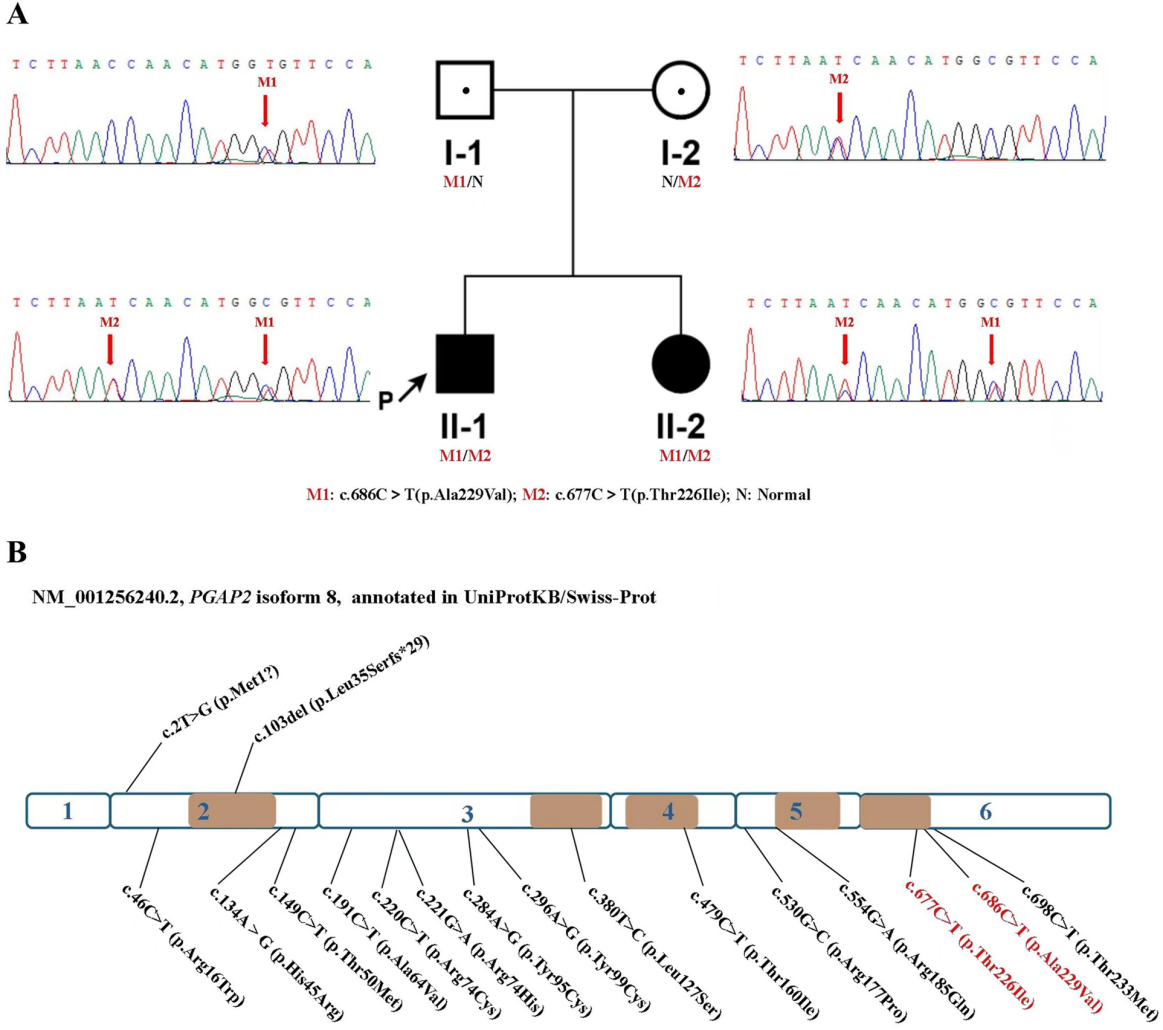
Two compound heterozygous variants of *PGAP2* were identified in this family, and up to now, a total of 17 variants of the *PGAP2* gene have been reported to be related to diseases. Variants are scattered throughout the protein without any specific domain. **(A)** Identification of two compound heterozygous mutations in *PGAP2*. Pedigree chart and Sanger Validation of the family. Individuals with the compound heterozygous mutations are represented with a full black square, and proven heterozygote carriers are shown by a dot. The proband is indicated with an arrow. **(B)** Schematic representation of the *PGAP2* gene. Exons 1-6 are depicted, truncting variants depicted above, non-truncting variants depicted below. The brown part indicates the transmembrane domain. The mutations of this study is marked in red.

Trio whole exome sequencing identified two missense variants in *PGAP2* gene [NM_001256240.2:c.686C>T (p.Ala229Val) and NM_001256240.2: c.677C>T (p.Thr226Ile)] in the proband, resulting in a compound heterozygous state, with the father and mother being heterozygous carriers of c.686C>T (p.Ala229Val) and c.677C>T (p.Thr226Ile) respectively. Sanger sequencing in the younger sister of the proband confirmed the existence of the two variants (Figure 3A). The c.686C>T (p.Ala229Val) is present in population databases at a very low frequency (rs753497329, max frequency is 0.004% in South Asian in gnomAD v4.0.0), the variant has been reported in a patient with *PGAP2*-related HPMRS (15). The c.677C>T (p.Thr226Ile) has been identified in only 1 heterozygote carrier within the gnomAD v4.0.0 database, and no patient with the variant has reported. Both the two variants are predicted to be deleterious by three in silico predictive software packages (PolyPhen-2, Mutation Taster, and SIFT). Moreover, both variants are located in the transmembrane domain (Figure 3B). Variant curation using ACMG/AMP guidelines suggests that the two variants are all classified as Variants of Uncertain Significance (PM2_Supporting + PM3_Supporting + PP1 + PP3 + PP4) (16).

*PGAP2* plays a critical role in the lipid remodeling steps of GPI-anchor maturation, essential for stable expression of GPI-anchored proteins at the cell surface (10). *PGAP2* gene encodes 16 different RNA transcripts, with 8 of which encode different isoforms and 8 that are noncoding RNAs. Among these, isoform 8(NM_001256240.2), which consists of 254 amino acids, is considered the biologically active isoform (2, 8, 15). This isoform has more than 90% homology with other mammalian orthologs (2). The UniProtKB/Swiss-Prot (18) annotate that it has five alpha-helix domains embedded in the Golgi membrane (Figure 3). To date, including cases from our patients, only 17 variants of this gene have been documented (Figure 3B, Table 1), predominantly characterized as missense mutations. Out of these, only two variants were truncating variants(c.103del [p.Leu35Serfs*90, and 2T > G (Met1?)] (9, 14) (Figure 3B, Table 1).

**Table 1:** Clinical and genetic characteristics of reported patients with *PGAP2*-related disorders.

Patient ID	Gender	Mutation	Central nervous system phenotypes				Auxiliary examinations			Facial features	Other findings	Reference
			DD/ID	epilepsy	Seizure semiology	Seizure control Yes/not	EEG	Brian CT/MRI	ALP			
Ⅱ-1	M	c.677C > T (p.Thr226Ile); c.686C > T (p.Ala229Val)	Globle developmental daley	Yes	epileptic spasms	Yes, positive response to ACTH treatment and high-dose pyridoxine.	interictal:hypsarrhythmia with burst suppression, ictal:clusters of spasms	MRI: thin corpus callosum, slightly enlarged lateral ventricles, mildly widened extracerebral spaces in bilateral fronto-temporo-parietal lobes	elevated, 789-3,380 U/L	thick eyebrows, hypertelorism in both eyes, and collapsed nasal bridge	hypotonia	This study
Ⅱ-2	F	c.677C > T (p.Thr226Ile); c.686C > T (p.Ala229Val)	Globle developmental daley	Yes	epileptic spasms	Yes, positive response to ACTH treatment and high dose pyridoxine.	interictal:hypsarrhythmia, ictal:epileptic spasms	MRI:hypoplasia corpus callosum	elevated, 619 U/L	thick eyebrows, collapsed nasal bridge, tented upper lip, and downward facing corners of the mouth	Large Mongolian patches on the dorsum and rump, hypotonia	This study
Patient 1	F	c.2T > G (p.Met1?); c.221G > A (p.Arg74His)	severe psychomotor retardation	Yes	epileptic spasms	Yes, positive response to ketogenic diet	hypsarrhythmia and suppression-burst	NA	elevated, 1,496-2,780 U/L	a flat occiput and pectus excavatum	low birth parameters, chest deformities in neonatal period; severe hypotonia, chronic fever, respiration insufficiency.	Jezela-Stanek et al. (14). Pronicka et al. (19).
individual A	F	c.46C > T (p.Arg16Trp); c.479C > T (p.Thr160Ile)	postnatal development, started to walk at the age of 18 months, her initial speech development was normal	Yes	Febrile seizures, tonic-clonic seizures	Yes, responded well to valproic acid	NA	NA	elevated, 2,107–2,448 U/L	broad nasal bridge, tented upper lip	-	Krawitz et al. (13)
individual B	M	c.380T > C (p.Leu127Ser); c.380T > C (p.Leu127Ser)	severe psychomotor developmently delay	Yes	Myoclonic, tonic-clonic seizures	Yes, responded well to AEDs	multifocal sharp waves	MRI: hypoplasia corpus callosum	elevated, 2,022 U/L	median cleft palate, wide palpebral fissures, a short nose with a broad nasal bridge, a tented upper lip, and a small jaw	Distal tapering of fingers and mild nail, hypoplasia of the fifth digit, hirschsprung disease, atrial septal defect, sensorineural hearing loss, microcephaly, scoliosis, severe muscular hypotonia	Krawitz et al. (13)
patient 1	F	c.103del (p.Leu35Serfs*29); c.134A > G (p.His45Arg)	Globle developmental daley, intellectual disability, speech delay	Yes	absence seizures	Yes, absence seizures were well-controlled on Ethosuximide; after pyridoxine and folinic acid supplementation, the patient continued to make developmental progress	runs of 4–6/s spike-wave complexes over the posterior half of the head in wakefulness and sleep, facilitated by eye closure, and photic stimulation at 16 Hz.	MRI:normal	elevated, 1,300–1,832 U/L	no dysmorphic features	hypotonia, precocious adrenarche, esotropia, myopic astigmatism	Messina et al. (9)
Patient 1(IV:4)	M	c.191C > T (p.Ala64Val); c.191C > T (p.Ala64Val)	intellectual disability, severely developmental regression	Yes	epileptic spasms	NA	no interictal epileptiform discharges, frequent spasms in ictal	NA	NA	NA	poor hearing, microcephaly	Naseer et al. (6)
Patient 2(IV:3)	F	c.191C > T (p.Ala64Val); c.191C > T (p.Ala64Val)	developmental delay, growth retardation, intellectual disability	Yes	epileptic spasms	NA	no interictal epileptiform discharges, frequent spasms in ictal	NA	NA	NA	poor hearing, microcephaly	Naseer et al. (6)
family MR043a(IV-1)	F	c.296A > G (p.Tyr99Cys); c.296A > G (p.Tyr99Cys)	severe intellectual disability, severe motor development delay	No	-	-	NA	CT: atrophy and increased gyration	elevated, 4,455 U/L	no major dysmorphisms	pronounced muscular weakness and hypotonia, strabismus, sleep disorders	Hansen et al. (2). Reuter et al. (20)
family MR043a(IV-2)	F	c.296A > G (p.Tyr99Cys); c.296A > G (p.Tyr99Cys)	severe intellectual disability, severe motor development delay	No	-	-	NA	CT: atrophy and increased gyration	elevated, 4,375vU/L	no major dysmorphisms	pronounced muscular weakness and hypotonia, strabismus, sleep disorders	Hansen et al. (2). Reuter et al. (20)
family MR043b(IV-4)	F	c.296A > G (p.Tyr99Cys); c.296A > G (p.Tyr99Cys)	severe motor development delay, speech delay	Yes	absence epilepsy	NA	NA	CT: brain atrophy	NA	no major dysmorphisms	severe muscular hypotonia, muscle biopsy identified muscle atrophy	Hansen et al. (2). Reuter et al. (20)
family MR5(V:4)	M	c.530G > C (p.Arg177Pro); c.530G > C (p.Arg177Pro)	intellectual disability	No	-	-	NA	MRI: normal	NA	normal	anemia	Hansen et al. (2).
family MR5(V:5)	M	c.530G > C (p.Arg177Pro); c.530G > C (p.Arg177Pro)	intellectual disability	No	-	-	NA	MRI: normal	NA	normal	anemia	Hansen et al. (2).
family MR5(V:6)	F	c.530G > C (p.Arg177Pro); c.530G > C (p.Arg177Pro)	intellectual disability	No	-	-	NA	MRI: normal	NA	normal	anemia	Hansen et al. (2).
family MR5(V:7)	F	c.530G > C (p.Arg177Pro); c.530G > C (p.Arg177Pro)	intellectual disability	No	-	-	NA	MRI: normal	NA	normal	anemia	Hansen et al. (2).
IV-3	M	c.554G > A (p.Arg185Gln); c.554G > A (p.Arg185Gln)	mild intellectual disability, speech difficulties	Yes	Past history of febrile seizures	NA	normal	CT: normal	elevated, 499 IU/L at 23 yrs of age	normal	mood problems, depression	Perez et al. (7)
IV-5	M	c.554G > A (p.Arg185Gln); c.554G > A (p.Arg185Gln)	developmental delay, Moderate intellectual disability	Yes	Past history of seizures without fever	NA	normal background activity, generalized spike and wave interictal discharges during wakefulness	MRI: normal	elevated, 673 IU/L at 17 yrs of age	normal	behavioral problems, aggression	Perez et al. (7)
IV-6	F	c.554G > A (p.Arg185Gln); c.554G > A (p.Arg185Gln)	mild intellectual disability, speech difficulties	No	-	-	NA	NA	elevated, >1,000 IU/L at 10 yrs of age	normal	mood problems, depression	Perez et al. (7)
IV-7	F	c.554G > A (p.Arg185Gln); c.554G > A (p.Arg185Gln)	developmental delay, mild intellectual disability, speech difficulties	No	-	-	NA	Normal spinal MRI	elevated, 1,318 IU/L at 10 yrs of age	normal	enuresis	Perez et al. (7)
Family 1(VI:4)	M	c.698C > T (p.Thr233Met); c.698C > T (p.Thr233Met)	Globle developmental daley	Yes	Unexplained major motor seizures	seizures had resolved without medication in old age	NA	NA	elevated, 2,440 IU/L	the characteristic facial gestalt remains characteristic throughout life, it generally becomes less pronounced in older patients	hypotonic, brachytelephalangy	Thompson et al. (8). Mabry et al. (21)
Family 1(VI:16)	M	c.698C > T (p.Thr233Met); c.698C > T (p.Thr233Met)	Globle developmental daley	Yes	Unexplained major motor seizures	seizures had resolved without medication in old age	NA	NA	elevated, 1,628 IU/L	The characteristic facial gestalt remains characteristic throughout life, it generally becomes less pronounced in older patients	hypotonic, brachytelephalangy	Thompson et al. (8).Mabry et al. (21)
Family 2(III-8)	F	c.698C > T (p.Thr233Met); c.698C > T (p.Thr233Met)	developmental disability	NA	NA	NA	NA	NA	NA	NA	NA	Thompson et al. (8).
Family 2(IV-2)	F	c.698C > T (p.Thr233Met); c.698C > T (p.Thr233Met)	developmental disability	NA	NA	NA	NA	NA	NA	NA	NA	Thompson et al. (8).
TF001_1	M	c.220C > T (p.Arg74Cys); c.220C > T (p.Arg74Cys)	Global developmental delay	NA	NA	NA	NA	NA	elevated, NA	NA	nystagmus	Froukh et al. (22)
TF001_4	M	c.220C > T (p.Arg74Cys); c.220C > T (p.Arg74Cys)	Global developmental delay	NA	NA	NA	NA	NA	elevated, NA	NA	nystagmus, aganglionic megacolon	Froukh et al. (22)
proband	M	c.149C > T (p.Thr50Met); c.686C > T (p.Ala229Val)	global developmental delay	Yes	focal seizures with impaired awareness	Treatment with high-dose pyridoxine showed partial benefit, ketogenic diet treatment.	progressive background activity disorganization, multifocal epileptic discharges.	MRI: mild hypoplasia of the inferior cerebellar vermis and corpus callosum and mild white matter reduction.	elevated, 1,005–1,769 U/L	normal	visual impairment	Saracino et al. (15)
patient	M	c.284A > G (p.Tyr95Cys) [along with *PGAP3* c.259G > A (p.Val87Met), heterozygote]	Global developmental/intellectual disability, delayed speech, delayed age at walking/no walking,	Yes	NA	NA	NA	NA	elevated, 3–4 times the upper limit of normal	short nose with broad tip	autistic features, hypotonia	Thompson et al. (23)

NA, not assessed; F, female; M, male; DD, developmental delay; ID, intellectual disability; EEG, electroencephalogram; MRI, magnetic resonance imaging; CT, computed tomography; ALP, alkaline phosphatase.

## Discussion

HPMRS-3 exhibits an autosomal recessive inheritance pattern, and individuals who are heterozygous carriers of variants typically do not display any symptoms. However, Yonatan et al. observed that heterozygous carriers may present with a mild phenotype characterized by slightly elevated serum levels of alkaline phosphatase and significant learning disabilities (7). We report two patients with HPMRS-3, who were diagnosed both clinically and genetically. In our cases, analysis of WES data revealed compound heterozygous mutations c.686C>T (p.Ala229Val) and c.677C>T (p.Thr226Ile) in *PGAP2* gene. The two variants are all classified as Variants of Uncertain Significance according to ACMG guidlines. Currently, it is difficult to classify the two variants as likely pathogenic or pathogenic variants because there have been few pedigrees hurboring the same variants reported, and missing functional experiments. However, using the ClinGen Bayesian classification framework (available at https://www.acgs.uk.com/quality/best-practice-guidelines/#VariantGuidelines), the variant got 5 points, which is very close to the criteria (6 points) for likely pathogenic variants. The two sibings' phenotypes matched the phenotype of *PGAP2*-related HPMRS, and the autosomal recessive pattern of segregation was observed in this family. After comprehensive consideration, the two patients can be diagnosed with *PGAP2*-related HPMRS-3.The c.677C > T (p.Thr226Ile) variants, identified as a novel disease locus, was initially reported to expand the genetic variation spectrum associated with HPMRS-3 and enhance our comprehension of its genetic basis.

When considering the location distribution of the missense mutations in *PGAP2*, approximately 1/3 are situated in the transmembrane domain, while the rest are found either within the Golgi lumen or in the cytoplasmic region (Figure 3B). Due to the limited number of mutations identified, it remains challenging to draw comparisons regarding the differences in clinical phenotypes that may arise from distinct mutation sites, as the sample size is too small to conduct a meaningful statistical analysis.

The core manifestations of HPMRS include hyperphosphatasia, seizures, and developmental disability, all of which were observed in both of our patients. Notably, persistent hyperphosphatasia distinguishes this disorder from other similar conditions and is consistently present in all patients with HPMRS. In our two patients, the levels of ALP ranged from 617 U/L to 3380 U/L. Seizures occur frequently throughout the course of the disease, manifesting in various forms such as absence, epileptic spasms, tonic-clonic, myoclonic, and febrile seizures (2, 6, 8, 9, 13, 14) (Table 1). The age of onset varies, ranging from the neonatal period to childhood (2, 6, 8, 9, 13–15). Epileptic spasms dominated the two children in our family during infancy, and atypical absence seizures occurred in the later stage of the proband. In the previously reported cases, epileptic spasms were infrequent, and some patients did not have seizures, however, epileptic spasms were typically the initial and predominant symptom in our two patients. As the third core features of HPMRS, severe or moderate psychomotor retardation was manifested in all patients. Most of them had no speech skills, and more than half of them could walk with or without a support (2, 6, 8, 9, 13–15). In our study, the patients exhibited global developmental delay, particularly evident in language and motor domains.

Other variable features such as microcephaly, hearing loss, strabismus, autism, behavior problem, and abnormal craniofacial features have also been reported (6, 7, 9, 13, 23) (Table 1). Mild facial deformities were observed in the patients from our two family. Their common expression is thick eyebrows, collapsed nasal bridge, at the same time there are hypertelorism in both eyes and a tent-shaped upper lip etc, which reported by the previous literature.Notably, distinct facial dysmorphisms have been documented in literature for several patients with HPMRS-3, including a girl exhibiting a broad nasal bridge and a tented upper lip, as well as a boy presenting with cleft palate, wide palpebral fissures, a short nose with a broad nasal bridge, a tented upper lip, and micrognathia (13). The observed facial dysmorphisms in HPMRS-3 patients resemble those seen in HPMRS patients with different genetic backgrounds (5, 24, 25). However, it is noteworthy that these facial dysmorphisms may attenuate with age (8, 13). It should be emphasized that genetic analysis is imperative for definitive diagnosis of HPMRS-3 and the presence of facial dysmorphisms is not obligatory.

Currently, there is no specific treatment available for HPMRS-3. Some patients achieve seizure control through monotherapy or combination therapy involving ASMs, pyridoxine and folinic acid, or ketogenic diet (9, 13, 14) (Table 1). In certain cases, seizures decrease in frequency over time and may even resolve without medication in older age (8). A case report described a girl with HPMRS-3 who experienced epileptic spasms and exhibited positive response to a ketogenic diet for drug-resistant epilepsy (14). In our Chinese family cohort, both cases mainly manifested epileptic spasms, accompanied by hypsarrhythmia of EEG, which were in line with the characteristics of infantile epileptic spasms syndrome (IESS), and thus were treated with ACTH. The positive responses of these two patients to the ACTH treatment further supported the notion that ACTH is the preferred treatment modality for epileptic spasms caused by various etiologies. For HPMRS-3 presenting with epileptic spasms, ACTH is a potentially effective anti-seizure medicine.

A study demonstrated that supplementation of pyridoxine and folinic acid can effectively correct low levels of PLP and 5-MTHFR in the cerebrospinal fluid of HPMRS-3 patients, suggesting the potential benefit of pyridoxine in managing refractory seizures in HPMRS-3 (9). Subsequently, in 2024, a patient diagnosed with HPMRS-3 also showed partial benefit with high-dose pyridoxine (15). Other studies have also indicated the potential benefits of pyridoxine supplementation in managing intractable seizures in HPMRS patients with other different genetic backgounds (15, 26–29). In our patient, pyridoxine supplementation was administered concomitantly with ASMs, resulting in well-controlled epilepsy but ineffective neurodevelopment. This indicates that further observation on a larger sample size is required to determine the efficacy of using pyridoxine for treating this disease.

## Conclusion

In conclusion, this case report represents the first documentation of *PGAP2*-related Hyperphosphatasia with impaired intellectual development syndrome in the Asian population, thereby expanding our understanding of the genetic and phenotypic spectrum of the disease. The patients in this family exhibited the typical clinical features of HPMRS, including elevated alkaline phosphatase (ALP), global developmental delay, seizures, and facial dysmorphisms. The diagnosis of HPMRS3 was confirmed through genetic testing, which revealed compound heterozygosity for variants in the *PGAP2* gene. The c.677c > T(p.Thr226Ile) variants expands the spectrum of PGAP2 gene variants associated with HPMRS3. The administration of ACTH and high-dose pyridoxine demonstrated efficacy in controlling seizures in the two patients, surpassing most previous cases. This finding holds significant clinical implications for the treatment of this disease. However, it should be noted that case reports and systematic studies related to *PGAP2* remain limited, warranting further research on the underlying pathogenic mechanisms and the development of standardized treatment strategies for *PGAP2*-related disorders.

## Data Availability

The data presented in the study are deposited in the Sequence Read Archive (SRA) data repository under the accession numbers SRR31480536, SRR31480535, and SRR31480534, which correspond to the proband, father, and mother, respectively.
